# Directly Observed Physical Activity of Year 1 Children during School Class Time: A Cross-Sectional Study

**DOI:** 10.3390/ijerph18073676

**Published:** 2021-04-01

**Authors:** Kirstin Macdonald, Nikki Milne, Rodney Pope, Robin Orr

**Affiliations:** 1 Physiotherapy Program, Faculty of Health Sciences and Medicine, Bond University, Gold Coast, Robina, QLD 4226, Australia; nmilne@bond.edu.au (N.M.); rpope@csu.edu.au (R.P.); rorr@bond.edu.au (R.O.); 2 School of Community Health, Charles Sturt University, Thurgoona, NSW 2640, Australia

**Keywords:** physical activity, direct observation, children, primary school, movement

## Abstract

Providing physical activity opportunities to children throughout the school day may be beneficial for children’s health and learning. Existing practices regarding the frequency, type and context of physical activity opportunities being provided to children in the early years of primary school remains largely unknown. The aim of this study was to observe Year 1 children’s physical activity and its contexts during school class time and identify opportunities to incorporate additional activity. A cross-sectional study was conducted with 34 Year 1 children (20 boys, 14 girls; mean age = 6.36 ± 0.34 years) from one primary school in Queensland, Australia. A modified version of the Observational System for Recording Physical Activity in Children—Elementary School was used to assess children’s physical activity and its contexts during class time. Observational data were collected over a four-week period. The frequencies (and percentages) of intervals of children’s activity observed in sedentary, light and moderate-to-vigorous intensities during different instructional and social contexts and physical settings were recorded and calculated. Pearson’s chi-square test of association was conducted to evaluate whether social context (group composition) was related to incidental physical activity. A total of 5305 observation intervals (i.e., 5 s observation interval followed by a 25 s recording interval) were available for analysis (~44 h of observation). Year 1 children were sedentary for the majority (86%) of observed intervals during school class time. Children spent limited time performing light (12% of intervals) and moderate-to-vigorous physical activity (2% of intervals). Organised physical activity observed during class time included physical education/school sport (5.9% of intervals) and classroom-based physical activity (2.8% of intervals). When children completed activities in small groups, they were significantly more likely to engage in incidental physical activity than when they completed activities as a whole class (χ^2^ = 94.73 *p* < 0.001). Incorporating movement into academic lessons or during transitions between lessons and classrooms may encourage children to be more active. Incidental physical activity may also be promoted through small group activities. Schools should ideally be encouraged and supported to employ a whole-of-school approach to physical activity promotion, which includes identifying and implementing opportunities for children to be active during class time.

## 1. Introduction

Overcoming low levels of physical activity (PA) among children and youth remains a public health priority globally, with recent figures suggesting the recommended 60 min of moderate-to-vigorous physical activity (MVPA) per day for optimal health are still not being adequately achieved [1]. A child or young person’s participation in regular PA is positively associated with numerous physical and mental health indicators [2,3,4,5,6], including cognition and academic performance [7,8]. Consequently, research evaluating the effectiveness of school-based PA interventions for improving children’s health and education outcomes has gained considerable momentum in recent years [7,8]. Although evidence to support beneficial effects of PA interventions on children’s cognition and overall academic performance remains inconclusive, strong evidence for beneficial effects on children’s mathematical outcomes has been reported [7,8]. Researchers have proposed a number of mechanisms that may underpin the PA-cognition relationship including biological, learning and psychosocial mechanisms, however hypotheses regarding these underlying mechanisms continue to be tested [9,10,11,12].

Healthy habits and behaviours are formed during the early childhood period [13]. Therefore, schools are ideally placed to positively influence children’s PA behaviour particularly during the early years of primary school [14,15]. For example, schools have been encouraged to employ a whole-of-school approach to plan, implement, and evaluate opportunities for children to be active throughout the school day through the development of a comprehensive school physical activity program (CSPAP) [16]. The provision of PA opportunities to students during the school day is considered a core component of a CSPAP. Organised PA during school class time has been defined as PA undertaken during physical education (PE) lessons, school sport and classroom-based PA and is exclusive of participation in PA during recess and lunch time [17]. Classroom-based PA involves: (i) integrating PA into academic lessons, (ii) providing PA breaks between lessons (with or without an academic focus), or (iii) incorporating PA into transitions from one location to another [16,17,18,19]. 

Several systematic reviews have reported beneficial effects of classroom-based PA interventions on the health (e.g., increased PA levels) [20,21] and education (e.g., classroom behaviour and academic performance) [18] outcomes of school students. However, notable limitations in the methodological quality of studies included in these reviews have been highlighted [18,20,21]. Despite these promising findings, to date there appear to be limited objective data available regarding how often classroom teachers are currently implementing classroom-based PA and which methods are being utilised [19,22].

During the early years of primary school, information regarding the frequency, type and exact context of children’s PA during class time is also limited [22]. Children’s PA in the school setting is commonly assessed using objective methods (e.g., accelerometry, pedometry, direct observation, and heart rate monitoring) and/or subjective methods (e.g., teacher, parent or self-report questionnaires or diaries) [23,24,25]. Whilst in recent years accelerometry and pedometry have been the most common methods of objectively measuring children’s PA [4,7,8,18], these methods are limited in their ability to capture the types and contexts of PA [23,26]. Direct (or systematic) observation may be the most suitable method for collecting information about the frequency, type and intensity of PA, whilst simultaneously recording information about the physical and social environment in which PA occurs, along with the educational context [23,26].

To inform the design of future school-based PA interventions, including classroom-based PA interventions, with children in the early years of primary school, it is necessary to determine existing practices regarding the frequency, type and context of PA opportunities being provided during class time. Observation of these practices will assist in identifying which PA opportunities may be the most realistic and practical for educators to incorporate into an already busy classroom schedule. Therefore, the aim of this study was to directly observe Year 1 children’s PA and the context of their PA during school class time and identify opportunities to incorporate additional activity. Based on the findings from the few studies which have investigated children’s PA across the primary school day using direct observation [22,27], it was hypothesized that Year 1 children would be predominantly sedentary during school class time, with limited opportunities to engage in physically active lessons and PA breaks.

## 2. Materials and Methods

### 2.1. Study Design

A cross-sectional research design was employed for this study and involved the collection of observational data over a four-week period. Ethics approval was obtained for the study from the Bond University Human Research Ethics Committee (Reference number: 15547). Research and gatekeeper approval were also granted by the south east region of the Queensland Department of Education (Reference number: 17/77163).

### 2.2. Setting and Participants

School principals from a representative selection of public, independent and Catholic primary schools in south east Queensland and northern New South Wales, Australia, were invited via email or telephone to involve their schools in the study, over the period from July to December 2017. The school principal at one public primary school in south east Queensland accepted the invitation and provided gatekeeper approval for four mainstream Year 1 classes to be involved. All children enrolled in the four Year 1 classes were invited to participate in the study. Information sheets and consent forms were circulated to the parents and guardians of 100 children from four Year 1 classes at the school. A recruitment goal of 40 participants was set, allowing a maximum of 10 participants to be selected from each Year 1 class. This number of participants was calculated to provide a margin of error of +/−15% for the population estimates derived from the sample of proportions of classroom time spent in different levels of PA, assuming a 95% level of confidence and a large underlying population of Year 1 children [28]. The high number of data points arising from observation of each additional child meant that observation of a larger number of participants, in order to further reduce the margin of error, was not feasible in the study context and within the available time frame. Written parental consent was obtained for 34 Year 1 children (*n* = 20 boys, *n* = 14 girls, mean age = 6.36 ± 0.34 years, range 5.42–7.25 years).

### 2.3. Outcome Measures

#### 2.3.1. Demographics

Age and sex were recorded for each participant and the Index of Community Socio-Educational Advantage (ICSEA) was noted for the school. ICSEA is a scale of socio-educational advantage calculated for Australian schools [29]. ICSEA values are set at an average of 1000 with an approximate range from 500 (schools with students with extremely educationally disadvantaged backgrounds) to 1300 (schools with students with very educationally advantaged backgrounds) [29]. A questionnaire was also completed by parents/caregivers regarding any relevant medical history for their child.

#### 2.3.2. Observational System for Recording Physical Activity in Children—Elementary School (OSRAC-E)

A modified version of the Observational System for Recording Physical Activity in Children-Elementary School (OSRAC-E) [27] was used to directly observe the participating Year 1 children’s PA in this study. The OSRAC-E is a direct observation tool designed to collect information about children’s PA within the primary school setting [27]. In addition to recording the intensity level and type of PA, the contextual and behavioral circumstances of children’s PA throughout the school day may be collected. This is in contrast to other direct observation tools, including the System for Observing Fitness Instruction Time (SOFIT) [30] and System for Observing Play and Leisure Activity in Youth (SOPLAY) [31], which assess children’s PA during physical education (PE) lessons and outdoor play, respectively. 

Direct observation is considered a valid and reliable method for assessing PA in children aged 3–18 years [23,26]. The OSRAC-E has been found to be a reliable direct observation system but has yet to be validated against other measures of PA [27,32]. However, to optimize reliability and validity in the present study, data were collected using the OSRAC-E in accordance with the recommendations developed by McKenzie and van der Mars for assessing children’s PA using systematic observation [26]. For example, prior to training, the observer (a registered physiotherapist) contacted the researchers who developed the OSRAC-E [27] to obtain the observation protocol, which included all category definitions and coding symbols. Advice was also sought regarding the most suitable software program to utilize to collect data electronically and subsequently the Multi-Option Observation System for Experimental Studies (MOOSES) software program [33] (Vanderbilt Kennedy Center, Nashville, TN, USA) was recommended. The observer reviewed the recommended training manual and undertook video observation and coding practice prior to live coding practice. As only one observer was involved in the study, interobserver reliability was not of concern. Finally, the developer of the MOOSES software program [33] was consulted to ensure correct use of the software program.

##### OSRAC-E Observation Protocol

The observation protocol for this study was based on that previously described by the researchers who developed the OSRAC-E [27]. The protocol involved observing one focal child at a time and used a momentary time-sampling procedure with a 5-s observation interval followed by a 25-s recording interval. A 20 min observation period was chosen for this study in accordance with the Year 1 class timetable, resulting in 40 observation intervals for the focal child who was being observed during each observation period. Each selected study participant was observed for approximately four 20 min periods (i.e., a total of 80 min per study participant). Observations were coded using the MOOSES software program [33] on a Microsoft Surface Pro tablet. For each observation session, the participant’s PA and its contexts were also recorded on a paper copy of the OSRAC-E, and where additional contextual information was required for the observed activity, qualitative information was noted.

##### Observation Categories and Codes

Observational information was collected, coded, and qualitatively documented across seven observational categories including; location, PA intensity level, PA type, physical setting, instructional setting, activity context, and group composition (for further information regarding the categories, codes and descriptions see McIver et al. [27]). Additional contextual information was also noted, including the time of day, reactivity to the presence of the observer, prompts for activity, information regarding who initiated the activity and whether transitions were directed by the teacher or incidental in nature. 

Several modifications to observation categories and codes were made to contextualize the OSRAC-E tool for the Australian primary school setting (Table A1). The *instructional setting* category was modified to include codes relating to the learning areas of the Year 1 Australian Curriculum, including the core (or priority) learning areas of English and mathematics [34]. The *other* code in the *instructional setting* category encompassed non-academic activities that were observed, including morning roll call, free play, show and share, meditation/mindfulness and organised school sport (excluding PE). The *activity context* category was also modified to include codes relating to classroom-based PA. The rationale for this modification was to allow for objective recording of the frequencies and types of classroom-based PA currently being provided to students in Year 1 classrooms. The coding of classroom-based PA was based on the definitions from the System for Observing Student Movement in Academic Routines and Transitions (SOSMART) [22]. For example, when classroom-based PA (excluding PE/school sport) was observed during school class time, it was noted whether these opportunities were *teacher-led* or *technology-led* (i.e., the teacher used technology, for example, online dance videos, to lead the activity) and whether there was an *academic* or *non-academic* focus to the activity. The *group composition* category of the OSRAC-E tool was also modified to record whether the class activity involved (i) the whole class engaged at the same time (e.g., all children sitting on the carpet listening to the teacher read a book) or (ii) small groups (e.g., children completing an activity while sitting at desks/on the carpet with or in the presence of a small group of peers).

Minor modifications were also made to the definitions of several types of transitions that were coded under observation categories. For the *instructional setting* category, the *change class* code was used when the teacher changed from one activity context to another. For example, when the corresponding *activity context* category was coded as *transition, snack break* or *classroom-based physical activity (non-academic)*. For the *activity context* category, the *transition* code was used when there was also a change from one activity context to another, however, this excluded *classroom-based PA* and *snack breaks,* as they were coded separately to better describe the nature of the transition. Transitions resulting in any light PA or MVPA for the focal child during academic lessons (i.e., excluding PE, school sport and classroom-based PA) were also recorded based on the definitions from the SOSMART [22]. For example, when teachers instructed the focal child to move from one area to another (e.g., from the carpet to desks) this was recorded as a *teacher-directed transition*. When the focal child moved without being instructed, this was recorded as an *incidental transition*.

### 2.4. Procedure

Observational data were collected over a four-week period during the second school term, from the end of May to the end of June 2018. The observations were conducted and recorded by a registered physiotherapist with experience of working with Year 1 children in the primary school setting. For each of the Year 1 classes, observation intervals occurred across one school week (i.e., Monday to Friday). All observation intervals took place during scheduled school class time (excluding recess and lunch breaks). Prior to observing each Year 1 class, the classroom teacher provided the observer with a copy of the class timetable. The observation intervals were then randomly selected from the class timetable using a random number generator to allocate four observation intervals for each participant from the available 20 min time periods across the school day. In cases where a participant was absent from class during their pre-planned observation interval, an alternative observation interval was allocated. To minimise student reactivity to the observer being present within the classroom, the teacher introduced the observer to the class on the Monday morning and observation intervals were not recorded during the first lesson, whilst the children adjusted to the observer being present. The classroom teacher and children in the class were not aware of exactly when observations occurred, and which study participant was being observed at any given time. Classroom teachers were advised to deliver their regular classroom curriculum, and to not modify their curriculum in any way due to the presence of the observer.

### 2.5. Statistical Analysis

Statistical analyses for this study were conducted using the Statistical Package for the Social Sciences (SPSS) (Version 26) [35]. Using the MOOSES software program [33], event frequencies and durations were calculated within each code group. The output from this process was then exported into an excel spreadsheet and SPSS analysis software (IBM Corporation, Chicago, USA). The number of observation intervals coded within each category was then calculated. In line with the previously published study using the OSRAC-E [27], PA intensity level was further coded and analysed as follows: Sedentary (stationary or limbs) = Level 1 code (stationary or motionless with no major limb movement or major joint movements) and Level 2 code (stationary with easy movement of limbs or trunk without translocation); Light PA = Level 3 code (translocation at a slow and easy pace); Moderate to vigorous PA = Level 4 code (translocation at a moderate pace) and Level 5 code (translocation at a fast or very fast pace) (see McIver et al. [27] for a detailed description of activity level codes). Cross-tabulation was used to calculate the numbers of intervals and percentages of total intervals observed in the different PA intensity levels by specific physical (i.e., location, physical setting), educational (instructional setting, activity context) and social (group composition) contexts. Pearson’s chi-square test of independence was used to evaluate whether group composition (i.e., ‘whole class’ or ‘group’) was related to PA intensity level. Pearson’s chi-square test of association was conducted to evaluate whether group composition was related to numbers of incidental transitions. To determine the effect size for the proportion of variance that was common to the two variables, Cramer’s V was calculated [36]. According to Cohen’s [37] conventions, an effect size ω of 0.1 can be considered small, 0.3 can be considered medium and 0.5 can be considered large [36]. A significance level of 5% (α = 0.05) was applied to all statistical tests.

## 3. Results

**Figure 1:**
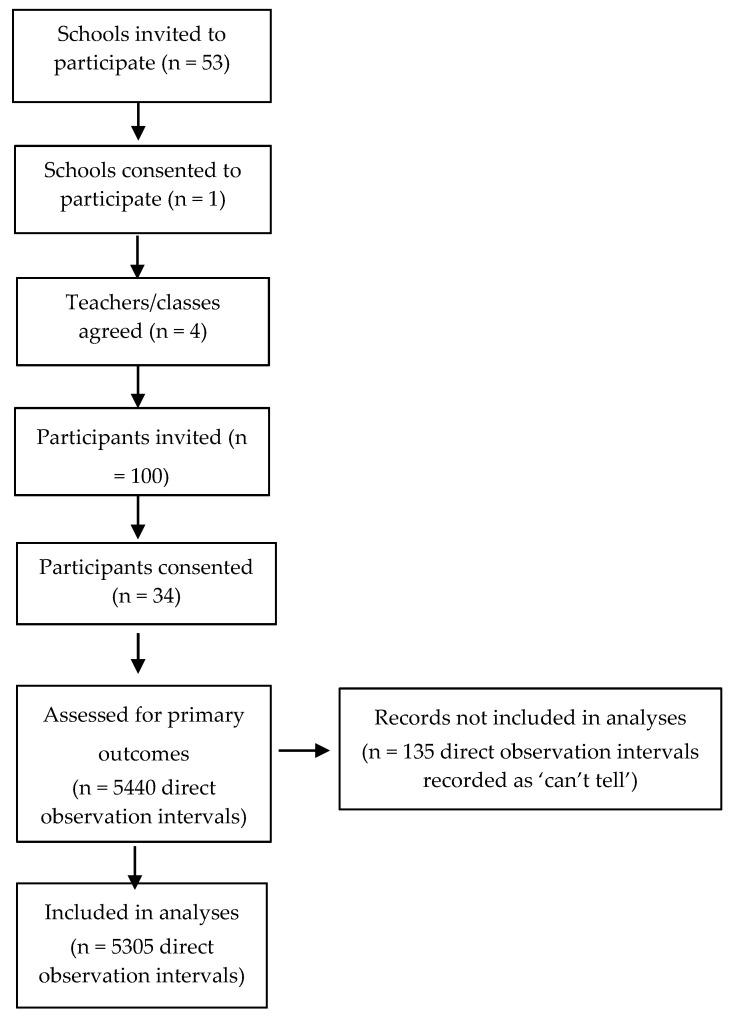
Flow of participants through study.

Data were collected for 34 study participants from four mainstream Year 1 classes at the cooperating Australian public primary school (*n* = 20 boys, mean age = 6.39 ± 0.23 years, range = 5.92–6.75 years; *n* = 14 girls, mean age = 6.31 ± 0.46 years, range = 5.42–7.25 years). The school ICSEA value was listed as 1059. Figure 1 summarises the flow of participants through the study. A total of 5440 observation intervals were recorded (i.e., 2 observations/min × 20 min period × 4 periods × 34 participants). Of these observation intervals, 135 were coded as ‘can’t tell’ for various categories due to participants moving out of the observer’s line of sight during indoor or outdoor activities. As such, a total of 5305 observation intervals were available for analysis following removal of these intervals.

The frequencies of observation intervals, including percentages of total observation intervals, occurring within each descriptive category for the observation intervals are presented in Table 1. The frequencies of observation intervals and percentages of observation intervals categorised as sedentary (i.e., level 1 and 2 codes), light PA (i.e., level 3 codes) and MVPA (i.e., level 4 and 5 codes) within each descriptive category are also presented in Table 1.

### 3.1. Location and Physical Setting

Overall, the majority of observation intervals of Year 1 participants during school class time occurred indoors within the classroom (92.5%) and these indoor intervals involved predominantly sedentary activities (88.4% sedentary, 11.1% light PA, 0.5% MVPA; Table 1). In contrast, Year 1 participants spent a considerably greater proportion of observation intervals engaged in light PA and MVPA when lessons were conducted outdoors on the sports field (light: 19.5%; MVPA: 19.8%).

### 3.2. Instructional Setting

The core learning areas of English and mathematics (72.2% of total intervals) represented the most common instructional contexts observed during school class time. The majority (88.8%) of intervals observed during core lessons involved sedentary activities, with minimal amounts of light PA (11%) and MVPA (0.2%) occurring. PE lessons were delivered by a specialist PE teacher once a week for a duration of 60 min and represented 4.4% of the total observed intervals. The types of activities observed during PE lessons targeted aerobic fitness (e.g., running, jumping) and motor skill development (e.g., throwing, catching, kicking). Consequently, participants undertook more light PA (16.7%) and MVPA (21.0%) during PE lessons than during core learning lessons, however, 62.2% of the observed intervals during PE lessons still involved activities classified as being sedentary. Close examination of the full set of PA intensity codes recorded for PE lessons (Table S1) revealed that 22.3% of observed intervals involved activities where participants were stationary with limb/trunk movement, consistent with activities such as standing while throwing, catching or kicking.

### 3.3. Activity Context and Activity Type

The majority of the observed intervals (81.8%) involved activities that were academic in nature and involved participants undertaking predominantly sedentary (88.7%) types of activities whilst sitting and standing. Of the academic learning areas of the Year 1 Australian Curriculum, participants were observed engaging in activities mainly relating to English (45.9%) and mathematics (24.7%). The regular class routine also comprised non-academic activities (e.g., morning roll call or free-time; 4.2% of total observed intervals) and short breaks where students were allowed to have fruit as a mid-morning snack.

Classroom-based PA (excluding PE and school sport) represented 2.8% of the total observed intervals. Classroom-based PA predominantly had a non-academic focus and was either delivered by the teacher (50 intervals or 0.9% of total observed intervals) or the teacher used technology (e.g., online dance video) to deliver the activity (77 intervals or 1.5% of total observed intervals). Classroom-based PA was mostly scheduled at times when teachers were transitioning students from one instructional context to another (*change class*). Academic content was seldom incorporated into classroom-based PA that was delivered by the teacher (10 intervals or 0.2% of total observed intervals) or delivered using technology (14 intervals or 0.3% of total observed intervals). Overall, children’s PA intensity levels during all types of classroom-based PA were classified as primarily sedentary (80.1%) with minimal light PA (11.3%) and MVPA (8.6%). However, examination of the full set of PA intensity codes recorded during classroom-based PA (Table S1) revealed that observed intervals involved slightly more stationary activities with limb/trunk movement (45.7%) than purely stationary activities (35.1%). This is consistent with the observation that the most common types of activities during classroom-based PA included standing and copying actions (43%), dancing (22.5%; e.g., copying a dance video), sitting while performing yoga (19.9%), walking around the classroom (5.3%), jumping/skipping (4.0%), movements while lying down (3.3%) and running on the spot (1.3%) (Table 2).

### 3.4. Group Composition

**Figure 2:**
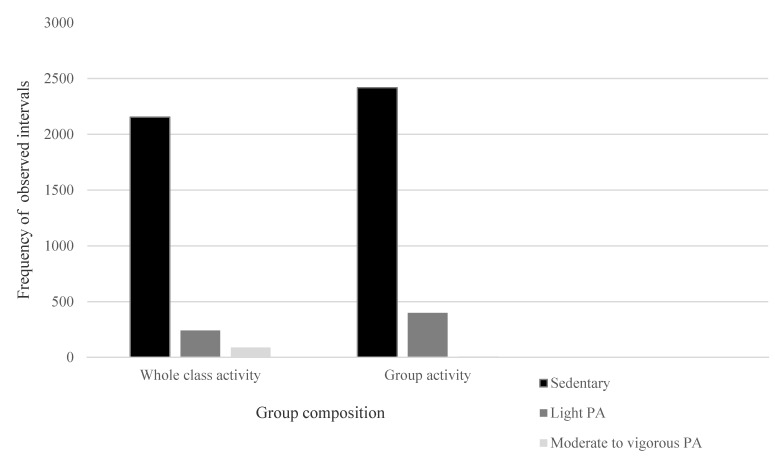
Frequencies of intervals observed in different intensity levels during whole class and group activities.

Classroom teachers used several different ways to group children during class activities. Nearly half (46.8%) of the observed intervals involved the whole class being engaged in activities at the same time. Just over a half (53.2%) of the intervals involved participants undertaking activities with or in the presence of a small group of peers (e.g., English/mathematics group rotations or sitting at desks with a group of peers). The Pearson’s chi-square test of independence, conducted to examine the relationship between group composition and levels of PA intensity, indicated a significant association between the two variables (χ^2^ = 98.98 *p* < 0.001) and so this relationship was further explored, graphically. Graphical representation of the observed intervals (Figure 2) indicates that when participants were involved in whole class activities, they were more likely to engage in MVPA, though MVPA remained relatively infrequent. Conversely, when participants completed activities in groups, with or in the presence of their peers, they were more likely to engage in light PA.

### 3.5. Transitions

**Figure 3:**
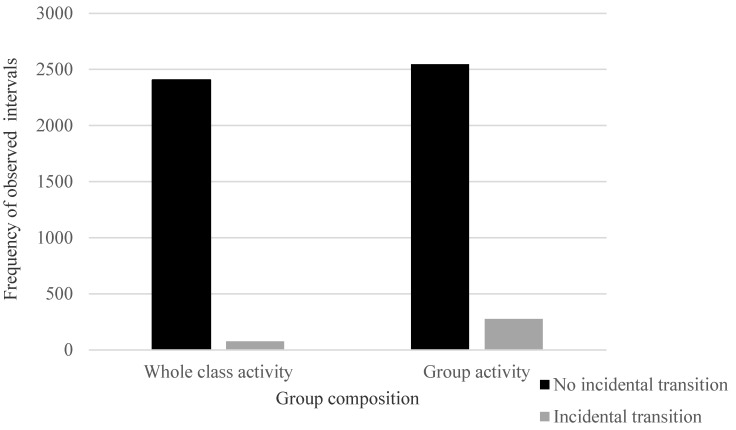
Frequency of incidental transitions observed during whole class and group activities.

In relation to the *location* category in the observations, *transitions* (e.g., from sports field to classroom) represented 1.1% of the total observed intervals and 25% of these transition intervals were sedentary (e.g., standing in line), 61.7% involved light PA (e.g., walking) and 13.3% involved MVPA (e.g., climbing stairs). In relation to the *instructional setting* category of observations, *change class* (i.e., indicating when the corresponding activity context category was coded as transition, snack break or classroom-based PA—non-academic) represented 5.6% of total observed intervals and 73.2% of these were sedentary, 21.8% involved light PA and 5.0% involved MVPA. In relation to the *activity context* category of observations, *transitions* (i.e., change in one activity context to another, excluding classroom-based PA and snack breaks) represented 2.5% of the total observed intervals and 63.1% of these were sedentary, 33.8% involved light PA and 3.1% involved MVPA (Table 1 and Table S1). The proportion of teacher-directed transitions and incidental transitions resulting in children undertaking light PA or MVPA (excluding observed intervals within PE, school sport and classroom-based PA—non-academic) was 4.2% and 6.7%, respectively (Table 1). The results of the Pearson’s chi-square test of association, conducted to examine the relationship between group composition and occurrence of incidental transitions, indicate that when children completed activities in small groups (with or in the presence of peers), they were significantly more likely to engage in incidental transitions than when they completed activities as a whole class (χ^2^ = 94.73 *p* < 0.001, ω = 0.134; Figure 3). However, teachers were not observed to direct children to move significantly more often during whole class activities than during group activities (χ^2^ = 1.31, *p* = 0.253).

## 4. Discussion

The aim of this study was to directly observe Year 1 children’s PA and the context of their PA during school class time and identify opportunities to incorporate additional activity. Overall, the study findings provide evidence that although Year 1 children are currently being provided with some occasional opportunities to be active during school class time, children were observed to be most frequently participating in academic activities that were sedentary in nature. Several opportunities to incorporate additional PA during school class time were identified. These included both structured (e.g., classroom-based PA) and unstructured (e.g., incidental movement) opportunities.

### 4.1. Existing PA Opportunities Provided to Year 1 Children during School Class Time

In Australia, the primary focus of learning in the early years of school is for children to develop essential skills in literacy and numeracy [38]. The focus on these core areas of learning in the Year 1 Australian Curriculum was evident in the present study as Year 1 participants engaged in academic activities primarily relating to English and mathematics for the majority of observation intervals. However, in line with other research conducted with children in the primary school setting [27,39], findings from this study revealed that the nature of these academic activities was predominantly sedentary. For example, in the study by McIver et al. [27], students in Kindergarten to Grade 5 were sedentary for the majority (84%) of observation intervals recorded using the OSRAC-E during the school day, with very few opportunities provided to children to accumulate MVPA throughout the school day.

In Australia it has been recommended that schools should ideally aim to deliver 150 min of organised PA to children each week [17]. It is noteworthy that in Australia, according to the Australian Curriculum, Assessment and Reporting Authority, the recommended time requirement for the subject of Health and Physical Education (comprised of two interrelated strands including personal, social and community health, and movement and physical activity) [40] is up to 80 h/year (2 h per school week); however, this policy is not mandatory [41]. In this study, Year 1 children were observed being provided with several opportunities to engage in organised PA during school class time, including planned PE lessons, a sports carnival (which replaced the weekly PE lesson) and classroom-based PA, but the time allocated was limited. Although it was not possible to quantify the exact duration of organised PA accumulated by Year 1 participants during class time across the whole school week, in the current study one 60 min PE lesson was scheduled into the school class timetable each week (of which an estimated 37.7% or 22 min of observed class time was spent undertaking light PA or MVPA). During the four-week study period, no additional sport time was timetabled, and observations (Table 1) indicated that non-PE organised PA (i.e., classroom-based PA) was limited to 2.8% of observed class time, with this equating to an estimated 18 min per week.

In relation to the intensity levels of PA undertaken by children, participants were observed to achieve higher levels of MVPA during organised PA opportunities than during classroom academic lessons. Year 1 children’s MVPA was most prevalent in PE lessons, with 21% of observation intervals during PE spent at this PA intensity. This finding is similar to that reported by McIver et al. [27], who found children from Kindergarten to Grade 5 spent 15% of observed PE lessons in MVPA. As outlined in a comprehensive school physical activity program, one of the indicators of quality PE is for children to spend 50% of PE lessons being active [16,42]. In the present study, sedentary activities coded during PE lessons were explained by periods when children were sitting or standing still, listening to instructions being given by the PE teacher, or waiting for their turn. Furthermore, observed PE lessons involved children engaging in gross motor skills such as catching, throwing and kicking, which meant that activities were coded as ‘stationary involving limb/trunk movement’ but with no translocation (Table S1). Therefore, providing professional development and training to classroom teachers and specialist PE teachers outlining methods to increase Year 1 children’s MVPA during PE lessons (e.g., incorporating higher intensity activities such as games that involve running and jumping) and strategies to minimise periods of inactivity (e.g., minimising transition times) may be important [42,43]. In future, it may be useful to validate the OSRAC-E against accelerometry to confirm whether MVPA coded using the OSRAC-E is correlated with levels of MVPA measured by accelerometry.

Although classroom-based PA was only observed on limited occasions (2.8% of the total time intervals), its presence during school class time provided evidence of how physically active lessons and PA breaks could be integrated into existing Year 1 class routines. The most popular types of classroom-based PA included PA breaks with a non-academic focus either delivered by the classroom teacher directly, or via the teacher using technology. Interestingly, the percentage of observed intervals that involved MVPA during teacher and technology-led PA breaks with a non-academic focus appeared to be higher than that observed during academic lessons, consistent with findings from experimental studies evaluating the impact of classroom-based PA interventions on children’s MVPA [44,45]. However, as there were only a small number of observed intervals involving classroom-based PA, these results should be interpreted with caution. The higher percentage of observed intervals involving children engaging in MVPA during whole class activities compared to group activities was most likely due to teachers structuring PE lessons and classroom-based PA opportunities as whole-class activities, and thus all children were encouraged to engage in these activities at the same time. This likelihood is supported by the supposition of Russ et al. [22] that classroom-based PA directed by a teacher may result in more MVPA than light PA, whereas incidental PA that occurs in the classroom may involve more light PA. Teacher and technology-led physically active academic lessons were seldom observed in the current study (<0.5% of total observed intervals), suggesting either that classroom teachers were not familiar with this approach in the classroom or that there may be barriers for teachers trying to integrate PA into academic lessons with Year 1 children. Overall, these findings were similar to those reported by Russ et al. [22], who reported during their pilot of the SOSMART tool that the median percentage of occurrence of non-academic and academic-infused movement within or between lessons was 2.2% (range 0–9.5%) and 0% (range 0–4%), respectively.

Interestingly, the majority of light PA recorded during observation intervals in the current study occurred during academic lessons. The most likely explanation for this was the number of teacher-directed and incidental transitions recorded during class activities or when changing classes from one instructional context to another, resulting in an accumulation of light PA. These findings are similar to those reported by Russ et al. [22], who observed a higher frequency of incidental types of movement than structured active lessons and breaks, in children aged seven to eight years old. Notably, the findings in the present study also indicate that when children completed classroom activities in small groups, with or in the presence of their peers, they were more likely to engage in light PA than when activities were completed as a whole class. This could be attributed to the fact that children were more freely able to move around the classroom to collect supplies or to talk to the teacher or their peers when working at their own pace during group-based activities. Conversely, when children were engaged in activities as a whole class, it often involved the teacher giving instructions to children while they sat on the carpet, which meant children were concentrating on listening to the teacher and were not required to move about the classroom to access supplies.

### 4.2. Future Opportunities to Incorporate Structured and Unstructured PA Opportunities into the Regular School Class Schedule

Evidence of the existing PA opportunities being provided to Year 1 children during school class time means that it may be possible to build upon this current practice. This study revealed that although classroom-based PA was seldom included during school class time, the most frequently used method was the inclusion of PA breaks during transitions from one instructional context to another, which may indicate this was relatively easy to implement into the class routine. Observed PA breaks typically had a non-academic focus and were either delivered by the classroom teacher or using technology (i.e., online dance videos). Classroom teachers were rarely observed incorporating movement into academic lessons, which suggests there is potential to further explore utilisation of this method. An array of resources have been developed to support teachers who wish to provide PA breaks and physically active lessons and these may be useful in assisting teachers to implement classroom-based PA more often during school class time (see review by Webster et al. [19] (p. 4) for some examples of resources available).

The types of activities that resulted in MVPA during classroom-based PA included running and jumping on the spot (Table 2). Dance videos primarily involved children standing on one spot while copying the corresponding movements. Unless activities such as jumping or running on the spot were repeated, most often they were coded as stationary with limb/trunk movement, due to there being no translocation. It would be important to validate the PA intensity levels achieved during dance videos, using accelerometry, to confirm the level of PA intensity children are undertaking. Further investigation is also warranted to determine whether to target specific intensities of PA (e.g., MVPA) during activities and/or whether children will benefit from any form of movement and breaking up sedentary time.

While classroom-based PA is one approach for teachers to more formally structure PA opportunities into the school day, findings from this study have shed light on the need for teachers and schools to consider the role the environment (e.g., the physical layout of the classroom, access to outdoor open spaces) and social context (e.g., class group composition) may play in increasing unstructured PA opportunities during school class time. The frequency of children’s incidental movement observed during classroom activities in the present study was related to the way the classroom teacher grouped children during those activities. This suggests that teachers may be able to influence the degree of children’s incidental PA in the classroom by scheduling group activities that may in turn lead to children moving around the classroom more often. Furthermore, structuring the physical layout of the classroom in a way that encourages children to move during classroom activities in order to collect supplies, communicate with others or interact with equipment or resources may also lead to an accumulation of incidental PA [19,22]. In addition, offering children a variety of different learning spaces and materials, for example, desks of different heights, may encourage children to regularly change position by kneeling, sitting or standing at different workstations during activities.

### 4.3. Study Limitations

It is important to acknowledge several limitations to this study. Firstly, the OSRAC-E direct observation tool has yet to be validated against other measures of PA such as accelerometry and thus recorded intensity levels of PA may have been over or underestimated. Research suggests that adopting multiple simultaneous approaches to measuring PA may lead to a more complete profile of children’s PA [23]. For this study, permission was initially sought to assess children’s PA using both accelerometry and direct observation to allow for this triangulation of data, however, approval to use accelerometers with study participants was not granted.

Another factor leading to a potential over or underestimation of PA intensity was that only a small number of participants were observed at one school, and these may differ from other children of the same age at the same or different schools. However, the observer did spend one whole school week with each Year 1 class over a four-week period and observe 34 different participants, and thus sampling was representative of the timetable (e.g., scheduled number of hours for English, mathematics, PE) and a range of children in the classes. Further limitations to the generalizability of the study findings include the fact that only one school agreed to participate, despite a more representative sample being invited. Nevertheless, given that teaching in all schools in Australia is guided by the Australian Curriculum [34], this observational study conducted in an Australian school provides a valuable indication of the extent to which PA may occur in Year 1 classrooms across many schools. To our knowledge, this study was the first to assess the frequency, type and context of Year 1 children’s PA in Australia using the OSRAC-E. Whilst further research of this nature with larger sample sizes is warranted, this study provides valuable insight into existing classroom routines and PA practices, as a guide and catalyst for further research.

It is important to also acknowledge that some of the observation intervals occurred during assessment weeks designed to facilitate mid-year reporting of student grades. This meant that in some activities such as English and mathematics small group rotations were not undertaken as planned, which may have resulted in an increase in sedentary time. However, these intervals represented less than 5% of the total number of observations.

Furthermore, the primary school setting is dynamic and thus observation of children’s PA was occasionally challenging, particularly during highly active periods when children were moving fast and there were many different activities occurring simultaneously. However, the advantage of the OSRAC-E being a focal child system meant that observer error due to environmental complexity was minimised, as long as the observer was able to view the focal child. Finally, all observation intervals were recorded by one observer. This may have subsequently resulted in observer bias leading to limitations in the generalizability of the findings. However, the observer had knowledge of, and experience in assessing children’s PA levels and motor proficiency, along with specific professional experience delivering gross motor programs to Year 1 students within the primary school context.

## 5. Conclusions

The collective findings from this study advance current understanding of Year 1 children’s PA and the context of this PA during school class time. The future opportunities available to incorporate PA into the regular class schedule were also identified. Overall, the findings reveal that Year 1 children were observed to be predominantly sedentary during school class time, undertaking limited amounts of light PA and MVPA, including organised and incidental PA. Implementing movement into academic lessons or during transitions between lessons was identified as a key strategy to encourage children to be more active during class time. Children’s incidental PA may also be facilitated by scheduling group activities and/or structuring the physical layout of the classroom to encourage movement. The findings from this study may interest school principals, classroom teachers, specialist PE teachers and other policy makers interested in identifying ways to implement opportunities for children in the early years of primary school to be active during class time, as part of a whole-of-school approach to PA promotion. Findings are also relevant to health professionals working in schools who are qualified to promote children’s health and wellbeing, as they may be able to support educators to implement these practices.

## Figures and Tables

**Table 1:** (i) Frequencies of observed intervals (and percentages of total intervals) in each category that were associated with each observation code; and (ii) frequencies of observed intervals (and percentage of intervals) in each category that were coded as sedentary, light and moderate-to-vigorous PA (adapted from McIver et al. [27]).

Observed Categories	Observed Codes	Observed Intervals (% Total)	Observed Intervals (%) by Physical Activity Intensity Level		
			Sedentary (Levels 1–2)	Light PA (Level 3)	Moderate-Vigorous PA (Levels 4–5)
	Total	5305 (100)	4570 (86.1)	639 (12.0)	96 (1.8)
Time of day	Morning	2465 (46.5)	2116 (85.8)	301 (12.2)	48 (1.9)
	Middle	2565 (48.4)	2220 (86.5)	298 (11.6)	47 (1.8)
	Afternoon	275 (5.2)	234 (85.1)	40 (14.5)	1 (0.4)
Location	Indoors	4906 (92.5)	4338 (88.4)	545 (11.1)	23 (0.5)
	Outdoors	339 (6.4)	217 (64.0)	57 (16.8)	65 (19.2)
	Transition	60 (1.1)	15 (25.0)	37 (61.7)	8 (13.3)
Physical activity type	Climb	11 (0.2)	11 (100)	0 (0)	0 (0)
	Crawl	22 (0.4)	0 (0)	21 (95.5)	1 (4.5)
	Dance	40 (0.8)	27 (67.5)	7 (17.5)	6 (15.0)
	Jump/skip	19 (0.4)	0 (0)	0 (0)	19 (100)
	Lie down	125 (2.4)	123 (98.4)	2 (1.6)	0 (0)
	Pull/push	29 (0.5)	23 (79.3)	1 (3.4)	5 (17.2)
	Run	59 (1.1)	0 (0)	0 (0)	59 (100)
	Sit/squat/kneel	3177 (59.9)	3177 (100)	0 (0)	0 (0)
	Stand	1187 (22.4)	1187 (100)	0 (0)	0 (0)
	Throw	22 (0.4)	22 (100)	0 (0)	0 (0)
	Walk	614 (11.6)	0 (0)	608 (99)	6 (1)
Physical setting	Classroom	4916 (92.7)	4349 (88.5)	544 (11.1)	23 (0.5)
	Hallway	50 (0.9)	15 (30.0)	29 (58.0)	6 (12.0)
	Sports field	339 (6.4)	206 (60.8)	66 (19.5)	67 (19.8)
Instructional setting	Change class *	298 (5.6)	218 (73.2)	65 (21.8)	15 (5.0)
	Core learning lessons *	3828 (72.2)	3399 (88.8)	421 (11)	8 (0.2)
	PE	233 (4.4)	145 (62.2)	39 (16.7)	49 (21.0)
	Languages	75 (1.4)	73 (97.3)	2 (2.7)	0 (0)
	Music	91 (1.7)	91 (100)	0 (0)	0 (0)
	Other *	355 (6.7)	301 (84.8)	36 (10.1)	18 (5.1)
	Science	130 (2.5)	124 (95.4)	6 (4.6)	0 (0)
	Technologies	40 (0.8)	24 (60)	14 (35)	2 (5)
	Visual arts	255 (4.8)	195 (76.5)	56 (22.0)	4 (1.6)
Activity context	Academics—Total	4337 (81.8)	3848 (88.7)	475 (11.0)	14 (0.3)
	Academics—English	2435 (45.9)	2183 (89.7)	247 (10.1)	5 (0.2)
	Academics—mathematics	1311 (24.7)	1158 (88.3)	150 (11.4)	3 (0.2)
	Classroom PA ^a^	151 (2.8)	121 (80.1)	17 (11.3)	13 (8.6)
	Classroom PA—Teacher-led (non-academic)	50 (0.9)	41 (82.0)	2 (4.0)	7 (14.0)
	Classroom PA—Teacher-led (academic)	10 (0.2)	10 (100)	0 (0)	0 (0)
	Classroom PA—Technology-led (non-academic)	77 (1.5)	60 (77.9)	11 (14.3)	6 (7.8)
	Classroom PA—Technology-led (academic)	14 (0.3)	10 (71.4)	4 (28.6)	0 (0)
	PE/school sport	313 (5.9)	192 (61.3)	56 (17.9)	65 (20.8)
	Non-academic	224 (4.2)	211 (94.2)	13 (5.8)	0 (0)
	Snack	124 (2.3)	92 (74.2)	32 (25.8)	0 (0)
	Transition *	130 (2.5)	82 (63.1)	44 (33.8)	4 (3.1)
	TV/video	26 (0.5)	24 (92.3)	2 (7.7)	0 (0)
Group composition	Whole class	2482 (46.8)	2153 (86.7)	241 (9.7)	88 (3.5)
	Group *	2823 (53.2)	2417 (85.6%)	398 (14.1)	8 (0.3)
Transitions	Teacher-directed Transition *	223 (4.2)			
	Incidental Transition *	353 (6.7)			

Classroom PA: classroom-based physical activity; PA: physical activity; PE: physical education. Values may not add up to exactly 100% due to rounding. Sedentary (stationary or limbs) = Level 1 code (stationary or motionless with no major limb movement or major joint movements) and Level 2 code (stationary with easy movement of limbs or trunk without translocation; Light PA = Level 3 code (translocation at a slow and easy pace); Moderate to vigorous PA = Level 4 code (translocation at a moderate pace) and Level 5 code (translocation at a fast or very fast pace) (^a^ See McIver et al. [27] for detailed description of activity level codes), * See Table A1 for definitions of modified OSRAC-E codes.

**Table 2:** Types of physical activity observed during classroom-based physical activity.

Observation Category	Observation Code	Observed Intervals (%)
Activity type	Total	151 (100)
	Climb	0 (0)
	Crawl	1 (0.7)
	Dance	34 (22.5)
	Jump/skip	6 (4.0)
	Lie down	5 (3.3)
	Pull/push	0 (0)
	Run	2 (1.3)
	Sit/squat/kneel	30 (19.9)
	Stand	65 (43.0)
	Throw	0 (0)

## Data Availability

Pending institutional and ethic board approval, the dataset analysed during the current study may be made available from the corresponding author on reasonable request.

## Supplementary Materials

The following are available online at https://www.mdpi.com/article/10.3390/ijerph18073676/s1, Table S1: Frequencies of observed intervals (and percentage of intervals) of physical activity opportunities provided during Year 1 school class time by physical activity intensity levels 1–5.

## Appendix A

**Table A1:** Description of modified codes for Observational System for Recording Physical Activity in Children-Elementary School.

**Observed Categories**	**Observed Codes ^a^**	**Description**
**Location**	Transition	When children move from an outdoor to an indoor location
Instructional setting	Change class	Teacher changed from one activity context to another (i.e., when the corresponding activity context category was coded as transition, snack break or classroom-based physical activity (teacher or technology-led with non-academic focus)
	Core learning lessons	English and mathematics. Inclusive of snack break and classroom PA (teacher or technology-led with academic focus)
	Other	Non-academic activities observed including morning roll call, free play, show and share, meditation/mindfulness, and school sport (excluding PE)
Activity context	Classroom PA	PA observed in classroom (excluding PE and school sport)
	Classroom PA—Teacher led (non-academic) ^b^	PA observed in classroom—delivered by the teacher, non-academic focus (i.e., PA break)
	Classroom PA—Teacher led (academic) ^b^	PA observed in classroom—delivered by the teacher, academic focus (i.e., active lesson)
	Classroom PA—Technology led (non-academic) ^b^	PA observed in classroom—delivered using technology (e.g., online dance video), non-academic focus
	Classroom PA—Technology led (academic) ^b^	PA observed in classroom—delivered using technology (e.g., online dance video), academic focus
	Physical education/school sport	Activity observed during PE lessons. School sport: the school sports carnival was timetabled into the regular 1-h PE lesson (i.e., replaced PE that one week)
	Snack	Eating fruit snack during break or while completing academic work
	Transition	Change in one activity context to another (excluding classroom-based PA and snack breaks)
Group composition	Whole class	Children complete an activity involving the whole class
	Group	Children complete an activity with or in the presence of a small group of peers
Transitions	Teacher-directed transition ^b^	The teacher asked students to move from one area to another (i.e., carpet to desk), either to start an activity (i.e., collect supplies), or finish an activity (i.e., pack up learning materials) in preparation for a new activity
	Incidental Transition ^b^	The student engaged in physical activity, without receiving instruction from the teacher to do so (i.e., collecting learning materials, moving to speak to the teacher/peers)

PA: physical activity; PE: physical education, ^a^ See McIver et al. [27] for original description of codes for observation categories, ^b^ See Russ et al. [22] for original description of codes for transitions.
