# Supplementary material for: Directly Observed Physical Activity of Year 1 Children during School Class Time: A Cross-Sectional Study
Source: Int J Environ Res Public Health. 2021 Apr 1;18(7):3676. doi: 10.3390/ijerph18073676 (PMC8036768; doi:10.3390/ijerph18073676)
Supplement: Supplementary file 1 [file ijerph-18-03676-s001.pdf]

Table S1: Frequencies of observed intervals (and percentage of intervals) of physical activity opportunities provided during Year 1 school class time by physical activity intensity levels 1-5

**Observation categories**	**Observation codes**	**Observed intervals (% total)**	**Observed intervals (%) by physical activity intensity level**						
			Stationary (level 1)	Limbs (level 2)	**Sedentary (levels 1 & 2)**	**Light (level 3)**	Moderate (level 4)	Fast (level 5)	**MVPA (levels 4 & 5)**
**Instructional setting**	Change class*	298 (5.6)	166 (55.7)	52 (17.4)	218 (73.2)	65 (21.8)	3 (1)	12 (4)	15 (5.0)
	Physical Education	233 (4.4)	93 (39.9)	52 (22.3)	145 (62.2)	39 (16.7)	0 (0)	49 (21)	49 (21)
**Activity context**	Classroom PA*	151 (2.8)	53 (35.1)	69 (45.7)	121 (80.1)	17 (11.3)	3 (2)	9 (10.2)	13 (8.6)
	Classroom PA Teacher-led (non-academic)*	50 (0.2)	21 (42.0)	20 (40)	41 (82)	2 (4)	1 (2.5)	6 (12)	7 (14)
	Classroom PA Teacher-led (academic)*	10 (0.2)	3 (30.0)	7 (70)	10 (100)	0 (0)	0 (0)	0 (0)	0 (0)
	Classroom PA – Technology-led (non-academic)*	77 (1.5)	28 (36.4)	33 (42.9)	60 (77.9)	11 (14.3)	2 (2.6)	3 (3.9)	6 (7.8)
	Classroom PA – Technology-led (academic)*	14 (0.3)	1 (7.1)	9 (64.3)	10 (71.4)	4 (28.6)	0 (0)	0 (0)	0 (0)
	PE + sport	313 (5.9)	128 (40.9)	64 (20.4)	192 (61.3)	56 (17.9)	2 (0.6)	63 (20.1)	65 (20.8)
**Group composition**	Whole class*	2482 (46.8)	1676 (67.5)	478 (19.3)	2153 (86.7)	241 (9.7)	6 (0.2)	81 (3.3)	88 (3.5)
	Group*	2823 (53.2)	1559 (55.2)	858 (30.4)	2417 (85.6)	398 (14.1)	1 (0.0)	7 (0.2)	8 (0.3)

Classroom PA: classroom-based physical activity; PA: physical activity; PE: physical education;
Values may not add up to exactly 100% due to rounding.
Sedentary (stationary or limbs) = Level 1 code (stationary or motionless with no major limb movement or major joint movements) and Level 2 code (stationary with easy movement of limbs or trunk without translocation; Light PA = Level 3 code (translocation at a slow and easy pace); Moderate to vigorous PA = Level 4 code (translocation at a moderate pace) and Level 5 code (translocation at a fast or very fast pace) (See McIver et al (2016) for detailed description of activity level codes)
*See Table A1 for definitions of modified OSRAC-E codes
